# Supplementary material for: Cross-basin and cross-taxa patterns of marine community tropicalization and deborealization in warming European seas
Source: Nat Commun. 2024 Mar 8;15:2126. doi: 10.1038/s41467-024-46526-y (PMC10923825; doi:10.1038/s41467-024-46526-y)
Supplement: Supplementary file 3 — Description of Additional Supplementary Files [file 41467_2024_46526_MOESM3_ESM.pdf]

Description of Additional Supplementary Files

Title: Supplementary Data 1

Description: Biodiversity time series datasets. Information of trends in sea temperature, CTIr, and underlying processes.
