# Supplementary material for: Cross-basin and cross-taxa patterns of marine community tropicalization and deborealization in warming European seas
Source: Nat Commun. 2024 Mar 8;15:2126. doi: 10.1038/s41467-024-46526-y (PMC10923825; doi:10.1038/s41467-024-46526-y)
Supplement: Supplementary file 5 — Reporting Summary [file 41467_2024_46526_MOESM5_ESM.pdf]

nature portfolio

Corresponding author(s): Guillem Chust

Last updated by author(s): Feb 9, 2024

Reporting Summary

Nature Portfolio wishes to improve the reproducibility of the work that we publish. This form provides structure for consistency and transparency in reporting. For further information on Nature Portfolio policies, see our Editorial Policies and the Editorial Policy Checklist.

Statistics

For all statistical analyses, confirm that the following items are present in the figure legend, table legend, main text, or Methods section.

n/a	Confirmed	
	x	The exact sample size (*n*) for each experimental group/condition, given as a discrete number and unit of measurement
	x	A statement on whether measurements were taken from distinct samples or whether the same sample was measured repeatedly
	x	The statistical test(s) used AND whether they are one- or two-sided<br>*Only common tests should be described solely by name; describe more complex techniques in the Methods section.*
	x	A description of all covariates tested
	x	A description of any assumptions or corrections, such as tests of normality and adjustment for multiple comparisons
	x	A full description of the statistical parameters including central tendency (e.g. means) or other basic estimates (e.g. regression coefficient) AND variation (e.g. standard deviation) or associated estimates of uncertainty (e.g. confidence intervals)
	x	For null hypothesis testing, the test statistic (e.g. *F*, *t*, *r*) with confidence intervals, effect sizes, degrees of freedom and *P* value noted<br>*Give P values as exact values whenever suitable.*
x		For Bayesian analysis, information on the choice of priors and Markov chain Monte Carlo settings
x		For hierarchical and complex designs, identification of the appropriate level for tests and full reporting of outcomes
x		Estimates of effect sizes (e.g. Cohen's *d*, Pearson's *r*), indicating how they were calculated

Our web collection on statistics for biologists contains articles on many of the points above.

Software and code

Policy information about availability of computer code

Data collection	Data collection has been undertaken with R language (R Core Team, 2022), OBIS (Ocean Biogeographic Information System; www.iobis.org), Global Ocean Data Assimilation System (GODAS) (www.cpc. ncep.noaa.gov/products/GODAS/), and MS EXCEL.<br>R Core Team (2022). R: A language and environment for statistical computing. R Foundation for Statistical Computing, Vienna, Austria. URL https://www.R-project.org/.
Data analysis	Data analysis have been undertaken in R Core Team (2022). R: A language and environment for statistical computing. R Foundation for Statistical Computing, Vienna, Austria. URL https://www.R-project.org/.<br>All R codes for data anaysis are available in a public repository (https://zenodo.org/records/10149019, DOI: 10.5281/zenodo.10149018).

For manuscripts utilizing custom algorithms or software that are central to the research but not yet described in published literature, software must be made available to editors and reviewers. We strongly encourage code deposition in a community repository (e.g. GitHub). See the Nature Portfolio guidelines for submitting code & software for further information.

Data

Policy information about availability of data

All manuscripts must include a data availability statement. This statement should provide the following information, where applicable:

- Accession codes, unique identifiers, or web links for publicly available datasets
- A description of any restrictions on data availability
- For clinical datasets or third party data, please ensure that the statement adheres to our policy

Biodiversity original data (i.e., species abundance at each year for each site survey) is subject to restrictions as it pertains to the corresponding institution. Certain original data is publicly available (DATRAS-ICES (2023), Danish marine monitoring (Josefson, and Rytter 2015), soft-bottom benthos in Basque estuaries (DOI: 10.5281/zenodo.10149018)) or from the corresponding author upon request.
Data generated during the study and that support their findings (all CTI time series, i.e. CTI per year for each site, and all underlying process scores ((de) tropicalization, (de)borealization) at per-species and per-site basis) are available in a public repository under accession code DOI: 10.5281/zenodo.10149018 (https://zenodo.org/records/10149019), and in Supplementary Data. Source data are provided with this paper.
DATRAS-ICES. ICES Database on Trawl Surveys (DATRAS), ICES, Copenhagen, Denmark. https://datras.ices.dk.) (2023).
Josefson A, Rytter D. Danish benthic marine monitoring data from ODAM. (ed Department of Bioscience - AU D) (2015). http://ipt.vliz.be/eurobis/

Research involving human participants, their data, or biological material

Policy information about studies with human participants or human data. See also policy information about sex, gender (identity/presentation), and sexual orientation and race, ethnicity and racism.

Reporting on sex and gender	The manuscript does not involve human participants, sex and gender.
Reporting on race, ethnicity, or other socially relevant groupings	The manuscript does not involve human participants, race, ethnicity or socially relevant groupings.
Population characteristics	The manuscript does not involve human participants.
Recruitment	The manuscript does not involve human participants.
Ethics oversight	The manuscript does not involve human participants, or manipulation or biological material, only data analysis.

Note that full information on the approval of the study protocol must also be provided in the manuscript.

Field-specific reporting

Please select the one below that is the best fit for your research. If you are not sure, read the appropriate sections before making your selection.

☐ Life sciences ☐ Behavioural & social sciences ☒ Ecological, evolutionary & environmental sciences

For a reference copy of the document with all sections, see nature.com/documents/nr-reporting-summary-flat.pdf

Ecological, evolutionary & environmental sciences study design

All studies must disclose on these points even when the disclosure is negative.

Study description	We compared the rate of temporal change in CTI (CTIr) and their underlying ecological processes in 65 biodiversity time series collected across the last four decades among six biological groups (hard-bottom and soft-bottom benthic communities, zooplankton, crustaceans, molluscs, and fish). Time series were collected in four areas within three European Seas (NE Atlantic, Mediterranean Sea and Baltic Sea), three distinct habitats (marine benthic or demersal, marine pelagic, and estuarine), and two basin types based on the presence or absence of dispersal barriers to ocean connectivity (non-enclosed sea, i.e. Atlantic Ocean, and semi-enclosed seas).<br>We compared the CTIr of marine communities across different factors and their levels. The comparison was based on linear mixed models of CTI with the interaction of year and factor as the fixed effect, the sampling sites as the random effect, and adding a temporal autoregressive function to test if the CTIr mean of a given level differs from zero. t- and p-values of the two-sided Wald test for the estimated coefficients are provided. To identify the most representative factors, we selected the best model using the Akaike's Information Criterion corrected (AICc) by comparing all combinations95. Diagnostic plots for the residuals of the selected factors were checked to ensure model reliability.
Research sample	We used 65 long-term time series corresponding to 1817 species taxonomically identified at species level (71 zooplankton species, 238 coastal hard-bottom benthic species, 923 coastal soft-bottom benthic species, 63 cephalopod species, 104 demersal crustacean species, 418 fish species) over varying durations during the last four decades (from 1980 to 2021 in the longest case). Time series ranges over varying durations during the last four decades (from 1980 to 2022 in the longest case). Most of time series span more than 15 years (92%), with median of 25 years, minimum of 9 years, and 20 and 39 years the 25% and 75% quartiles, respectively. Detailed information on each time series such as sampling procedures, time series length, biological community, and location is provided in Supplementary Information 1.<br>Sample size is the relative abundance of species at annual basis in order to match with sea temperature data.

Biodiversity time series datasets. Information on sampling procedures and time series.

1 Demersal fish in the North-East Atlantic (DATRAS)

Freely available long-term monitoring data on marine fish communities from six bottom-trawl surveys were downloaded from DATRAS-ICES (the Database of Trawl Surveys). The bottom-trawl surveys included the North Sea (NS-IBTS), Baltic Sea (BITS) and the Scottish (SWC-IBTS) International Bottom Trawl Surveys, as well as the French Surveys (EVHOE) in the NE Bay of Biscay . All surveys use standardized sampling protocols, where bottom trawls are towed for an average of 30 min and the species composition and abundances of all captured fishes are identified and recorded to the finest taxonomic level possible. The spatial coverage and resolution differed across surveys, and we therefore aggregated trawl surveys to 1x1° spatial grid cells. The length of time series also differed between surveys, and we therefore examined the period 1980–2015, which maximized temporal overlap between surveys. The arithmetic mean of each single species abundance was calculated for each year on each ecoregion. Within each region, only grid cells with > 20 years observations were selected for further analysis.

2 Demersal and benthic fish, cephalopods, and crustaceans in the Western Mediterranean (MEDITS)

Data on demersal and benthic fish, cephalopods, and crustaceans was collected by the MEDITS program (Spedicato et al. 2020) which started in 1994 in the Mediterranean with the cooperation among research institutes from four countries: France, Greece, Italy and Spain . MEDITS consist of series of bottom trawl surveys that routinely provide abundance indices of target species for tuning stock assessment models of intermediate complexity (Spedicato et al. 2019). The data to calculate the CTI in this study include annual means of species abundance (km m-2) of demersal fish and benthic crustaceans and cephalopods sampled from May to July during the 1994-2019 period in the western Mediterranean Sea, specifically within Geographical Sub Area 6 (GS6) as established by General Fisheries Commission for the Mediterranean. The analysis was carried out in three subregions of the GS6 area: (i) North at > 41° latitude north (North of Ebro Delta), (ii) Centre within 41-39.2° latitude north and (iii) South at < 39.2° latitude north. The division of these three areas show different ocean warming velocities.

3 Pelagic, demersal and benthic fish, cephalopods, and crustaceans in the Western Mediterranean (PUR/TRA)

Data on abundance and composition of fish, crustaceans and cephalopods during the 2000-2020 period were obtained using purse-seine and bottom trawl daily catch data from different ports and fishing gear across the northern Catalan coast. The data was divided into three regions: (i) north including ports within 41.6° latitude north, (ii) central at 41.2° latitude north and (iii) south including data around 40.6° latitude north.

Daily data on landings span the 2000-2020 period and come from 23 different fishing ports scattered along the Catalan Sea. Raw data were grouped by species, gear, port and year. Here, we exclusively considered main fishing gears in the area: purse-seiners and bottom trawlers (hereafter trawlers). Purse-seiners operate through the water column and target pelagic fish species (mostly anchovy Engraulis encrasicolus and sardine Sardina pilchardus). Therefore, their catches can mainly inform about the pelagic fish community. Bottom trawlers operate near the bottom and integrate the benthic and demersal community. Whereas purse-seiners mainly target pelagic fish and few cephalopod species, landings from trawlers are taxonomically diverse, including many species of fish and invertebrates. Here, we considered main groups of species for trawlers: fish, crustaceans, and cephalopods. Trends in CTI were evaluated by aggregating daily landing data into annual values. Fishing ports were grouped within three main subregions: North, Central and South.

4 Demersal and benthic fish, cephalopods, and crustaceans in the Eastern Mediterranean

A large bottom trawling fishery-independent time series data provided by the Israel fishery Department was used in the analysis. The targeted groups included demersal fish, crustaceans, and cephalopods in the 31-50 m. Surveys took place onboard bottom trawlers with fishing the Israeli continental shelf, between latitudes 31º20' N and 33º05' N. Depths ranged between 15 and 300 m. The catch sampling protocol (Edelist et al. 2011) from 1990 to 1994 was repeated in all subsequent periods. The data used in this analysis covers a period from the early 1990s to recent years with some gaps mostly in the first two decades. The units used in the CTI analysis are individuals per sampling box or haul.

5 Fish in Wadden Sea (FYKE)

The Royal Netherlands Institute for Sea Research (NIOZ) collects data on the Wadden Sea fish community using traditional fixed gear at a specific location (Lat 52.997ºN, Long 4.775ºE) since 1960. The fixed gear is known as a 'kom-fyke' (van Leeuwen et al. 2023) . It is a passive fish trap consisting of a 200-m net running from the beach towards deeper waters. Fish captured each day are identified to species level. To carry out the CTI analysis, the average yearly catch per species was corrected for effort (number of fishing days) and the data were filtered for the period 1980-2021.

6 Fish in Gironde estuary

The Gironde estuary, located SW France is one of the largest European estuaries (Lobry et al. 2003). Its surface area is approximately 625 km2 at high tide. The dataset comes from the Blayais Power Plant monitoring program. In the frame of this program, fish sampling surveys are conducted monthly since the late 1970s. Data from 1985 was selected since the sampling protocol is considered stable and standardized from this date. Three sampling sites are located along four transects. On each site, simultaneously, one fishing sample is taken near the surface and one near the bottom. Surface samples are taken using two 4.0 x 1.0 m rectangular frame nets fitted both sides of the boat. Details in the sampling protocol can be found in Lobry et al. (2006) and in Chevillot et al. (2017). The monitored fauna consists mainly of small fish species and juveniles of larger species. In total, 47 species are considered in the analysis. They can be divided into 4 ecological guilds (Marine M, Freshwater FW, Diadromous DIA and Estuarine Resident ER) following (Franco et al. 2008) and (Courrat et al. 2011) (Dethier et al. 1993). In this case study, the CTI was calculated by averaging fish abundance (i.e., the number of individuals per 1,000 m3 of filtered water) across the twelve sites (i.e., 3 sites and 4 transects) for each year.

7 Fish in the Pertuis Charentais nursery ground

The Pertuis Charentais (SW France) are a complex ensemble that includes the estuary of the Seudre and Charente rivers, as well as their discharges in two semi-enclosed bays (Pertuis Breton and Pertuis d'Antioche) sheltered by two islands (Ile de Ré and Ile d'Oléron). This 540 km2 macrotidal embayment serves as a major nursery ground for juvenile marine fish in the Bay of Biscay. Here, we analysed a 9-year time-series of fish abundance data collected by Ifremer (see for instance: (Le Pape et al. 2003, Trimoreau et al. 2013)) during late summer-early autumn, between 1997 and 2019, using a 2.9 m wide and 0.5 m high scientific beam trawl with a 20 mm stretched mesh size in the cod end. Trawls were conducted during the day for 15 minutes at a mean speed of 2.5 knots.

8 Fish in Basque estuaries

A network of monitoring trawl lines along the 12 main Basque estuaries, from the inner, middle, and outer reaches (three to five trawl lines, per estuary), was established by the Basque Government, from which we selected 4 estuaries with extensive intertidal flats and lowest historical anthropogenic pollution (Barbadun, Lea, Urola, Butroe) (Borja et al. 2016). The demersal assemblage sampling was carried out every September – October, at high tide, between 2002 and 2022, once every 3 years at each of the estuaries. According to Hemingway and Elliott (2002), at each of these trawl lines, three hauls (replicates) were collected, using a 1.5 m wide beam trawl with a tickler chain; the first part of the net has 10 mm mesh size and 8 mm mesh size cod end; and towed for 10 min at 1.5 knots (sometimes the trawl period might differ, when rocks or other obstacles made the trawling difficult). Site locations were initially determined by the suitability of the seabed for trawling sampling as well as by the requirement to incorporate the whole of the salinity range within each of the estuaries. Finally, fish density was calculated considering fishing effort (beam width, time of trawling and boat speed). Samples were identified and counted on-board immediately. Species which could not be identified were fixed in a solution of 4% ethanol, then examined in the laboratory (Uriarte and Borja 2009). The mean of each single species abundance was calculated for each year.

9 Hard bottom benthic intertidal communities in UK coastline

MarClim survey intertidal invertebrates and macroalgae species. Surveys are in areas of extensive, exposed intertidal rocky reef or artificial, hard, coastal structures/defences away from areas of coastline that are heavily developed or utilized for social or economic purposes, and avoiding riverine and estuarine outputs. Rocky intertidal surveys for MarClim are in SACFOR scale and represent species cover in quadrats (Burrows et al. 2020). The four ecoregions analysed here were UK Southwest, North Wales, South Wales and UK South) consist of time series data including sites >15 years along the UK and Ireland coasts during 2002-2020.

10 Hard-bottom coralligenous communities in the Western Mediterranean

Coralligenous assemblages represent a mosaic of different habitats home to calcareous algae and invertebrates such as corals, sponges, bryozoans or tunicates. Long-term ecological data on coralligenous assemblages was obtained from two monitoring stations (replicates, i.e., Petit Conglue and Grotte Pères) established in Calanques Natural Park (Marseille, France) in 2008 at a depth of 18-22 m. Photographs of the two sites were first analysed separately at three different years (i.e., 2018; 2012 and 2020), and then pooled together into single annual values representative for the Calanques location. Overall, 48 photographic quadrats (sub-replicates) of 25 x 25 cm (24 quadrats per site) were analysed. The sampling unit (625 cm2 per quadrat) was selected following previous studies in the same communities (e.g., Gómez-Gras et al. (2021)). The percent cover of the different subtidal macro-benthic sessile species was calculated in each quadrat by over-imposing 100 stratified random points and identifying the underlying species to the lowest possible taxonomic level, using Photoquad software (Trygonis and Sini 2012).

11 Hard-bottom benthos in the Ligurian Sea

Data on rocky benthos was collected along the coast of the Island of Capraia (43.048 N, 9.828 E), about 40 miles off the west coast of Italy, in the Ligurian Sea (northwest Mediterranean). Low intertidal assemblages (0 and −0.3 m above mean low water level) are dominated by belts of the canopy-forming fucoid Ericaria amentacea (previous name Cystoseira amentacea Bory var. stricta Montagne) which alternate with patches occupied by algal turfs, encrusting coralline algae or bare rock (Bulleri et al. 2002, Tamburello et al. 2013), varying in size between tens to hundreds of cm2.

Assemblages were sampled annually in summer, from 2007 to 2016, in 64 contiguous quadrats, 50 x 50 cm in size. The abundance of sessile macroalgae and invertebrates was assessed visually in 20 x 20 cm quadrats that were placed in the center of each 50 x 50 cm quadrat along each transect. The final percentage covers were calculated by summing over the 25 sub-quadrats (Dethier et al. 1993). Data were classified at the species level.

12 Soft and hard-bottom macrobenthic communities in the Bay of Biscay

Hard-bottom macrobenthic community: Intertidal rocky benthic communities including hard bottom macroinvertebrates, lichens and macroalgae were analysed during 2002-2020 within “Littoral Water Quality Monitoring and Control Network”, from the Basque Water Agency (URA) (Borja et al. 2016). Samples were collected every 3 years in 26 intertidal transects along the Basque coast, perpendicular to the shoreline. A semi-quantitative sampling of surface coverage similar to Braun-Blanquet3 was carried out across the transects, scaled from 1 (low coverage) to 7 (high coverage). The arithmetic mean of each single species abundance was calculated for each year.

Soft-bottom macrobenthic community: Subtidal soft-bottom macroinvertebrates were sampled with a van Veen grab (0.1 m2) at ~30 m depth and identified to species level to estimate abundance (individuals/m2) and community composition at all stations along the Basque coast within “Littoral Water Quality Monitoring and Control Network”, from the Basque Water Agency (URA) (Borja et al. 2016). The sampling of most of the stations started in 1995. At each station, an annual sample was taken in winter, consisting of 3 replicates. The data used in the calculation of CTI includes stations with the lowest anthropogenic impact. The arithmetic mean of each single species abundance was calculated for each year.

13 Soft-bottom benthos in the Kattegat

The Danish soft-bottom benthic macrofauna in the Kattegat Sea was divided into Northwest and Southwest areas according to a salinity gradient observed in the region (Sildever et al. 2015). 85% of the stations analyzed were sites with >15 years within the 1980-2012 period. Subtidal soft-bottom macroinvertebrates samples were taken from surface to 60 m depth. Freely available data contains abundance (number of individuals per species) and biomass (wet weight or dry weight per species) information (Josefson and Rytter 2015) . We used number of counts per species across the Southwest and Northwest areas in the CTI analysis.

14 Soft-bottom benthos in Wadden Sea

Intertidal benthic macroinvertebrates were sampled at 15 sampling stations in the Balgzand tidal flat (Western Wadden Sea). Samples were taken yearly in March from 1970 until 2019. Only data from 1980 onwards were used for this study. Twelve of the sampling stations consisted of a 1 km transect in which 50 equally spaced core samples of 0.018 m2 were taken to a depth of 30 cm. Three more sampling stations consisted in squares of 900 m2 were nine randomly positioned cores of 0.1 m2 plus nine cores of 0.01 m2 were taken. All cores were sieved with a 1 mm mesh and animals caught were identified and counted in the lab. Abundance values in terms of individual per m2 for each year were used in the CTI analysis across the sampling stations. A more detailed description of the methods can be found in (Beukema 1974, Beukema and Cadée 1997).

15 Soft-bottom benthos in western Chanel (L4)

Starting in 2008, the Plymouth Marine Laboratory benthic survey is an ongoing ecological survey within the Western Chanel Observatory. Monthly benthic biological sampling is undertaken at a variety of sites within the Plymouth Sound, focusing primarily on the marine biodiversity reference site, station L4. L4 is an exposed site with a depth of approximately 54 m, with sediments ranging from mud to sand. For this research, we have used subtidal benthic macroinvertebrates data collected using 0.1 m2 box-cores deployed from the Plymouth Marine Laboratory research vessel Plymouth Quest and separated using a 0.5 mm mesh. For each sampling occasion 4 replicates are taken. Abundance is presented as individuals per m2. In the CTI analysis, the mean of each single species abundance for each year was used.

16 Zooplankton across European seas

We included time series data of copepods across the North Atlantic and Mediterranean Sea (Urdaibai, Saronikos, Kattegat), previously reported in Villarino et al. (2020), as well as the L4 for zooplankton in the western English Channel. Year means of abundance in terms of individuals per m3 were used in the CTI analysis across the four time-series.

The Kattegat is a sub-area of the North Sea (56.95° N, 11.30° E), which is a transition zone between the Baltic Sea and the North Sea, with a substantially higher salinity range than the 2 sea areas it connects. The mean depth is ~20 m with a maximum depth > 90 m at the northern boundary, and half the area is shallower than 25 m (Matthews et al. 1999).

The estuary of Oka, in Urdaibai (43° 22' N, 2°43'W) is a temperate estuary located on the Basque coast in the southern Bay of Biscay. It is a relatively short (12.5 km), shallow (mean depth of 3 m), meso-macrotidal and marine-dominated system, with high salinity waters in the outer half and a stronger axial gradient of salinity towards the head (Villate et al. 2008). The zooplankton series used in this study corresponds to the monthly sampling at high tide in the salinity zone of around 35 located at the mouth of the estuary, which is inhabited by neritic zooplankton (Fanjul et al. 2018).

The Gulf of Saronikos is a semi-enclosed embayment on the western coastline of the Aegean Sea, in the eastern Mediterranean Sea. Saronikos Station 11 (Saronikos S11) is in the Saronikos Gulf at 37° 52.36' N, 23° 38.30' E with a bottom depth of 78 m. Mesozooplankton sampling was performed by vertical hauls (WP2 net, 200 µm) from ~75 m to the surface.

Station L4 is a well-established European coastal time-series station located in the western English Channel (Eloire et al. 2010). Samples are collected weekly by vertical net hauls (WP2 net, mesh 200 µm) from 50 m to the surface (Atkinson et al. 2015). Sea floor depth is 54 m and most of the water column zooplankton are sampled by these 0-50 m hauls (Parry et al. 2020). Abundance is presented as numbers per m3. Here, year means of individual per m3 over 1988-2017 period were used for the CTI analysis in the L4 case study (Atkinson et al. 2015).

References

Atkinson, A., R. A. Harmer, C. E. Widdicombe, A. J. McEvoy, T. J. Smyth, D. G. Cummings, P. J. Somerfield, J. L. Maud, and K. McConville. 2015. Questioning the role of phenology shifts and trophic mismatching in a planktonic food web. Progress in Oceanography 137:498-512.

Beukema, J. J. 1974. Seasonal changes in the biomass of the macro-benthos of a tidal flat area in the Dutch Wadden Sea. Netherlands Journal of Sea Research 8:94-107.

Beukema, J. J., and G. C. Cadée. 1997. Local differences in macrozoobenthic response to enhanced food supply caused by mild eutrophication in a Wadden Sea area. Food is only locally a limiting factor. Limnology and Oceanography 42:1424-1435.

Borja, Á., G. Chust, J. G. Rodríguez, J. Bald, M. J. Belzunce-Segarra, J. Franco, J. M. Garmendia, J. Larreta, I. Menchaca, I. Muxika, O. Solaun, M. Revilla, A. Uriarte, V. Valencia, and I. Zorita. 2016. 'The past is the future of the present': Learning from long-time series of marine monitoring. Science of the Total Environment 566–567:698-711.

Bulleri, F., I. Bertocci, and F. Micheli. 2002. Interplay of encrusting coralline algae and sea urchins in maintaining alternative habitats. Marine Ecology Progress Series 243:101-109.

Burrows, M. T., S. J. Hawkins, J. J. Moore, L. Adams, H. Sugden, L. Firth, and N. Mieszkowska. 2020. Global-scale species distributions predict temperature-related changes in species composition of rocky shore communities in Britain. Global Change Biology 26:2093-2105.

Chevillot, X., H. Drouineau, P. Lambert, L. Carassou, B. Sautour, and J. Lobry. 2017. Toward a phenological mismatch in estuarine pelagic food web? Plos One 12:e0173752.

Courrat, A., R. P. Dominguez, M. Elliott, M. Lepage, A. Borja, A. Uriarte, J. Neto, and V. Raykov. 2011. Wiser WP4. 4: Fish in transitional

waters, data presentation. Page 15 in Wiser Uncertainty Workshop.
Dethier, M. N., E. S. Graham, S. Cohen, and L. M. Tear. 1993. Visual versus random-point percent cover estimations: 'objective' is not always better. Marine Ecology Progress Series 96:93-100.
Edelist, D., O. Sonin, D. Golani, G. Rilov, and E. Spanier. 2011. Spatiotemporal patterns of catch and discards of the Israeli Mediterranean trawl fishery in the early 1990 s: ecological and conservation perspectives. Scientia Marina(Barcelona) 75:641-652.
Eloire, D., P. J. Somerfield, D. V. P. Conway, C. Halsband-Lenk, R. Harris, and D. Bonnet. 2010. Temporal variability and community composition of zooplankton at station L4 in the Western Channel: 20 years of sampling. Journal of Plankton Research 32:657-679.
Fanjul, A., A. Iriarte, F. Villate, I. Uriarte, A. Atkinson, and K. Cook. 2018. Zooplankton seasonality across a latitudinal gradient in the Northeast Atlantic Shelves Province. Continental Shelf Research 160:49-62.
Franco, A., M. Elliott, P. Franzoi, and P. Torricelli. 2008. Life strategies of fishes in European estuaries: the functional guild approach. Marine Ecology Progress Series 354:219-228.
Gómez-Gras, D., C. Linares, A. López-Sanz, R. Amate, J. B. Ledoux, N. Bensoussan, P. Drap, O. Bianchimani, C. Marschal, O. Torrents, F. Zuberer, E. Cebrian, N. Teixidó, M. Zabala, S. Kipson, D. K. Kersting, I. Montero-Serra, M. Pagès-Escolà, A. Medrano, M. Frleta-Valić, D. Dimarchopoulou, P. López-Sendino, and J. Garrabou. 2021. Population collapse of habitat-forming species in the Mediterranean: a long-term study of gorgonian populations affected by recurrent marine heatwaves. Proceedings of the Royal Society B: Biological Sciences 288:20212384.
Hemingway, K. L., and M. Elliott. 2002. Field Methods. Pages 410-509 Fishes in Estuaries.
Josefson, A., and D. Rytter. 2015. Danish benthic marine monitoring data from ODAM.
Le Pape, O., F. Chauvet, S. Mahévas, P. Lazure, D. Guérault, and Y. Désaunay. 2003. Quantitative description of habitat suitability for the juvenile common sole (Solea solea, L.) in the Bay of Biscay (France) and the contribution of different habitats to the adult population. Journal of Sea Research 50:139-149.
Lobry, J., M. Lepage, and E. Rochard. 2006. From seasonal patterns to a reference situation in an estuarine environment: Example of the small fish and shrimp fauna of the Gironde estuary (SW France). Estuarine, Coastal and Shelf Science 70:239-250.
Lobry, J., L. Mourand, E. Rochard, and P. Elie. 2003. Structure of the Gironde estuarine fish assemblages: a comparison of European estuaries perspective. Aquatic Living Resources 16:47-58.
Matthews, J. B. L., F. Buchholz, R. Saborowski, G. A. Tarling, S. Dallot, and J. P. Labat. 1999. On the physical oceanography of the Kattegat and Clyde Sea area, 1996–98, as background to ecophysiological studies on the planktonic crustacean, Meganyctiphanes norvegica (Euphausiacea). Helgoland Marine Research 53:70-84.
Parry, H. E., A. Atkinson, P. J. Somerfield, and P. K. Lindeque. 2020. A metabarcoding comparison of taxonomic richness and composition between the water column and the benthic boundary layer. ICES Journal of Marine Science 78:3333-3341.
Sildever, S., T. J. Andersen, S. Ribeiro, and M. Ellegaard. 2015. Influence of surface salinity gradient on dinoflagellate cyst community structure, abundance and morphology in the Baltic Sea, Kattegat and Skagerrak. Estuarine, Coastal and Shelf Science 155:1-7.
Spedicato, M. T., E. Massutí, B. Mérigot, G. Tserpes, A. Jadaud, and G. Relini. 2019. The MEDITS trawl survey specifications in an ecosystem approach to fishery management. Scientia Marina 83:9-20.
Spedicato, M. T., E. Massutí, B. Mérigot, G. Tserpes, A. Jadaud, and G. Relini. 2020. The MEDITS trawl survey specifications in an ecosystem approach to fishery management. Scientia Marina 83:9.
Tamburello, L., F. Bulleri, I. Bertocci, E. Maggi, and L. Benedetti-Cecchi. 2013. Reddened seascapes: experimentally induced shifts in 1/fspectra of spatial variability in rocky intertidal assemblages. Ecology 94:1102-1111.
Trimoreau, E., B. Archambault, A. Brind'Amour, M. Lepage, J. Guitton, and O. Le Pape. 2013. A quantitative estimate of the function of soft-bottom sheltered coastal areas as essential flatfish nursery habitat. Estuarine, Coastal and Shelf Science 133:193-205.
Trygonis, V., and M. Sini. 2012. photoQuad: A dedicated seabed image processing software, and a comparative error analysis of four photoquadrat methods. Journal of Experimental Marine Biology and Ecology 424-425:99-108.
Uriarte, A., and A. Borja. 2009. Assessing fish quality status in transitional waters, within the European Water Framework Directive: Setting boundary classes and responding to anthropogenic pressures. Estuarine, Coastal and Shelf Science 82:214-224.
van Leeuwen, A., H. van der Veer, and J. I. J. Witte. 2023. NIOZ fyke programme Stuifdijk. NIOZ.
Villarino, E., X. Irigoien, F. Villate, A. Iriarte, I. Uriarte, S. Zervoudaki, J. Carstensen, T. D. O'Brien, and G. Chust. 2020. Response of copepod communities to ocean warming in three time-series across the North Atlantic and Mediterranean Sea. Marine Ecology Progress Series 636:47-61.
Villate, F., G. Aravena, A. Iriarte, and I. Uriarte. 2008. Axial variability in the relationship of chlorophyll a with climatic factors and the North Atlantic Oscillation in a Basque coast estuary, Bay of Biscay (1997–2006). Journal of Plankton Research 30:1041-1049.

Sampling strategy	Sample size is the relative abundance of species at annual basis. In terms of spatial units, sample size depends on biological group and location. See Supplementary Information 1.
Data collection	Data collection of each time series used in the study is summarised in Supplementary Data and available in a public repository (https://zenodo.org/records/10149019, DOI: 10.5281/zenodo.10149018).<br>Data collection has been undertaken with R language (R Core Team, 2022), OBIS (Ocean Biogeographic Information System; www.iobis.org), Global Ocean Data Assimilation System (GODAS) (www.cpc. ncep.noaa.gov/products/GODAS/), and MS EXCEL.<br>R Core Team (2022). R: A language and environment for statistical computing. R Foundation for Statistical Computing, Vienna, Austria. URL https://www.R-project.org/.<br>Authors Ernesto Villarino and Guillem Chust collected abovementioned data and biodiversity data provided by some authors.
Timing and spatial scale	Time period of each time series used in the study is indicated in Supplementary Data.
Data exclusions	No data were excluded
Reproducibility	A set of R-language codes has been used for the analysis and available in a public repository (https://zenodo.org/records/10149019, DOI: 10.5281/zenodo.10149018) in order to ensure the reproducibility of the results.
Randomization	CTIr and their underlying ecological processes in biodiversity time series were grouped according to 6 biological groups (hard-bottom and soft-bottom coastal benthic communities, zooplankton, demersal crustaceans, cephalopods, and fish), 4 areas within three European Seas (NE Atlantic, Mediterranean Sea and Baltic Sea), 3 distinct habitats (marine benthic or demersal, marine pelagic, and estuarine), and 2 basin types based on the presence or absence of dispersal barriers to ocean connectivity (non-enclosed sea, i.e.

	Atlantic Ocean, and semi-enclosed seas). Random allocation was not possible as depends on historic monitoring programs. The covariation of factors was controlled with model selection using AICc.
Blinding	Blinding was not used. We consider this is not applicable to our study since the analysis was undertaken without excluding any time series. We first compiled biodiversity time series available from the research groups with long time span (>9 years) and a minimum of 3 sampled years, and subsequently we undertook the analysis of those data series.

Did the study involve field work? ☒ Yes ☐ No

Field work, collection and transport

Field conditions	Temperature associated to each biodiversity time series is indicated in Supplementary Data.
Location	Biodiversity time series are located across European seas (see Figure 2a and Supplementary Data for geographic coordinates). Water depth information for each site study is provided in Supplementary Information 1.
Access & import/export	The study used existing census data of wild marine organisms.
Disturbance	The study used existing census data of wild marine organisms.

Reporting for specific materials, systems and methods

We require information from authors about some types of materials, experimental systems and methods used in many studies. Here, indicate whether each material, system or method listed is relevant to your study. If you are not sure if a list item applies to your research, read the appropriate section before selecting a response.

Materials & experimental systems

n/a	Involved in the study	
☒	☐	Antibodies
☒	☐	Eukaryotic cell lines
☒	☐	Palaeontology and archaeology
☐	☒	Animals and other organisms
☒	☐	Clinical data
☒	☐	Dual use research of concern
☐	☒	Plants

Methods

n/a	Involved in the study	
☒	☐	ChIP-seq
☒	☐	Flow cytometry
☒	☐	MRI-based neuroimaging

Animals and other research organisms

Policy information about studies involving animals; ARRIVE guidelines recommended for reporting animal research, and Sex and Gender in Research

Laboratory animals	The study did not involve experiments in animals in laboratory.
Wild animals	The study used existing census data of wild marine animals (fish, invertebrates).
Reporting on sex	The study did not considered sex in the study design.
Field-collected samples	The study used existing data of field-collected marine animals (fish, invertebrates).
Ethics oversight	No ethical approval was required on the study protocol since the study used existing data

Note that full information on the approval of the study protocol must also be provided in the manuscript.

Dual use research of concern

Policy information about dual use research of concern

Hazards

Could the accidental, deliberate or reckless misuse of agents or technologies generated in the work, or the application of information presented in the manuscript, pose a threat to:

No	Yes	
☒	☐	Public health
☒	☐	National security
☒	☐	Crops and/or livestock
☒	☐	Ecosystems
☒	☐	Any other significant area

Experiments of concern

Does the work involve any of these experiments of concern:

No	Yes	
☒	☐	Demonstrate how to render a vaccine ineffective
☒	☐	Confer resistance to therapeutically useful antibiotics or antiviral agents
☒	☐	Enhance the virulence of a pathogen or render a nonpathogen virulent
☒	☐	Increase transmissibility of a pathogen
☒	☐	Alter the host range of a pathogen
☒	☐	Enable evasion of diagnostic/detection modalities
☒	☐	Enable the weaponization of a biological agent or toxin
☒	☐	Any other potentially harmful combination of experiments and agents
